# Down‐regulation of miR‐21‐5p by pirfenidone to inhibit fibroblast proliferation in the treatment of acquired tracheal stenosis

**DOI:** 10.1111/crj.13727

**Published:** 2023-12-27

**Authors:** Wentao Li, Pingping Huang, Jinmei Wei, Sen Tan, Guangnan Liu, Qiu Yang, Guangfa Wang

**Affiliations:** ^1^ Department of Respiratory and Critical Care Medicine The Second Affiliated Hospital of Guangxi Medical University Nanning China; ^2^ Department of Ophthalmology Ruikang Hospital Affiliated to Guangxi University of Chinese Medicine Nanning China; ^3^ Department of Respiratory and Critical Care Medicine Peking University First Hospital Beijing China

**Keywords:** acquired tracheal stenosis, cell proliferation, fibrosis, MiR‐21‐5p, pirfenidone

## Abstract

**Objective:**

Treatment options for acquired tracheal stenosis (ATS) are limited due to a series of pathophysiological changes including inflammation and cell proliferation. Micro ribonucleic acid‐21‐5p (miR‐21‐5p) may promote the excessive proliferation of fibroblasts. However, various types of fibrosis can be prevented with pirfenidone (PFD). Currently, the effect of PFD on miR‐21‐5p and its biological function has not been clarified. In this study, PFD was evaluated as a potential treatment for ATS by inducing fibroblast proliferation in lipopolysaccharide (LPS)‐induced fibroblasts by targeting miR‐21‐5p.

**Methods:**

For 48 h, 1 g/ml LPS was used to generate fibroblasts in vitro, followed by the separation of cells into four groups: control, PFD, mimic, and mimic + PFD. The Cell Counting Kit‐8 (CCK‐8) technique was adopted to measure the proliferation of fibroblasts. Real‐time quantitative polymerase chain reaction (RT‐qPCR) and Western blot (WB) were used to measure the relative expressions of tumor necrosis factor‐α (TNF‐α), transforming growth factor‐β1 (TGF‐β1), drosophila mothers against decapentaplegic 7 (Smad7) and collagen type I alpha 1(COL1A1) messenger RNA (mRNA) and proteins, respectively.

**Results:**

(1) At 0, 24, 48, and 72 h, fibroblast growth was assessed using the CCK‐8 method. Compared with the control group, the mimic group showed the highest fibroblast viability, and the PFD group showed the lowest fibroblast viability. However, fibroblast viability increased in the mimic + PFD group but decreased in the mimic one. (2) RT‐qPCR and WB showed that the mimic group exhibited a significant up‐regulation in the relative expressions of TNF‐α, TGF‐β1, and COL1A1 mRNA and proteins but a down‐regulation in the relative expression of Smad7 mRNA and protein compared with the control one. In the PFD group, the results were the opposite. Nevertheless, the relative expressions of TNF‐α, TGF‐β1, and COL1A1 mRNA and proteins were increased, whereas that of Smad7 mRNA was decreased in the mimic + PFD group. The change was less in the mimic group.

**Conclusion:**

PFD may have a preventive and curative effect on ATS by inhibiting fibroblast proliferation and the fibrotic process and possibly through down‐regulating miR‐21‐5p and up‐regulating Smad7 and its mediated fibrotic and inflammatory responses.

## Graphical abstract

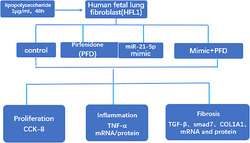

## 1 INTRODUCTION

Tracheostomy and prolonged intubation are the most common causes of benign acquired tracheal stenosis (AST).^1^ Under the influence of pathogenic factors, pathological repair occurs in the healing process after trachea injury. It involves a series of complex pathophysiological changes, including inflammation, cell differentiation and proliferation, extracellular matrix (ECM) accumulation, and tissue structure reconstruction. These pathophysiological changes are manifested as the excessive proliferation of effector cells dominated by fibroblasts and the massive deposition of ECMs dominated by type I and III collagen, which eventually leads to tracheal stenosis.^2^, ^3^ Currently, respiratory interventional therapy, which includes thermal ablation, balloon dilation, cryotherapy, and stent implantation, is the primary treatment option for traumatic airway stenosis.^4^ In addition to the surgical resection of the narrow tracheal segment, the end‐to‐end anastomosis of the tracheal is applied. However, airway restenosis will still occur no matter whether medical interventions or surgical operations may become secondary injuries. To treat tracheal stenosis effectively, it is necessary to prevent aggravation and pathological repair as early as possible.

As one of the most abundant and highly conserved micro ribonucleic acids (miRNAs), miR‐21 is located in the vacuole membrane protein 1 (VMP1) gene on chromosome 17 and functions to regulate target gene expression. Among them, miR‐21‐5p promotes fibroblast proliferation excessively through pro‐inflammatory,^5^ pro‐fibrotic,^6^ and apoptosis‐inhibiting effects,^7^ which contributes to the increased synthesis of large amounts of collagen, the accumulation of ECMs, and the formation of fibrous connective tissues. This results in excessive airway repair and scar proliferation after injury and aggravates local and systemic inflammatory responses in the airway by the up‐regulation of transforming growth factor‐1 (TGF‐1)‐mediated expression and the release of inflammatory factors. The result is airway stenosis.^8^, ^9^ In contrast, pirfenidone (PFD) is a pyridone‐derived pleiotropic molecular drug and the first drug approved by the Food and Drug Administration (FDA) for treating idiopathic pulmonary fibrosis. Due to its extensive anti‐fibrotic, ‐inflammatory, and ‐oxidant effects, PFD is now gradually being utilized in skin scarring,^10^ postoperative ocular scarring,^11^ pulmonary,^12^ renal s,^13^ and liver fibrosis^14^ and other treatments with good efficacy. What is the effect of PFD on miR‐21‐5p and its biological function? This study intended to explore the effect and mechanism of miR‐21‐5p on lipopolysaccharide (LPS)‐induced fibroblast proliferation by PFD intervention, to provide a strong experimental basis for the clinical drug treatment of post‐injury airway stenosis.

## 2 METHODS

### 2.1 Grouping of cell experiments and the preparation of working solutions

1. The experiments were divided into four groups: control (wild type), PDF (1 mmol/L PDF), mimic (miR‐21‐5p mimic), and mimic + PDF groups (miR‐21‐5p mimic + PDF).
2. The mimetic powder of miR‐21‐5p first underwent 5‐min centrifugation at 12 000 rpm at 4°C to ensure that the sample settled to the bottom.
3. Then, 125 μL of double‐distilled water (ddH2O) was added, respectively, and dissolved fully to obtain a final concentration of 2.5 nmol and stored in a −20°C refrigerator for backup.

### 2.2 Transient cell transfection

1. Human fetal lung fibroblast 1 (HFL1) cells were observed. Their growth state was induced with 1 μg/mL LPS. HFL1 cells were digested and counted with trypsin. Each well was inoculated in a 6‐well plate with a density of 2 × 106 cells/mL. After that, 2 mL per well was added until the cells were fused to about 65–75%.
2. The complete medium in the 6‐well plate was changed to 2 mL/well without double antibodies and continued to incubate in a cell culture incubator for 1 h before transfection.
3. Then, 3.75 μL of Lipofectamine TM 3000 reagent and 125 μL of Ham's F‐12K medium were taken in a sterilized 1.5‐mL EP tube, mixed thoroughly, and left for 5 min at room temperature.
4. Finally, 125 μL of Ham's F‐12K medium and 3 μL of plasmid small interfering RNS (siRNA) were mixed in an autoclaved 1.5‐mL EP tube and made stand at room temperature for 5 min.
5. Steps (3) and (4) were mixed gently and made stand at room temperature for 20 min.
6. The 96‐well plate was removed. Next, 250 μL of the mixture in step (5) was added to each well and shaken gently to mix well. The cells underwent 48‐h transfection in the incubator, and RNA was extracted for subsequent experiments.

### 2.3 Drug concentration screening of PFD

1. HFL1 cells at the stage of logarithmic growth were digested with a trypsin digestion solution and then planted in 6‐well plates at 2 × 106 cells/mL, followed by the addition of 2 mL to each well and incubation in a 5% carbon dioxide (CO_2_) cell incubator at 37°C. A control group and different concentrations of PFD groups were set up;
2. According to the literature, PFD concentrations of 0, 0.5, 1, 1.5, and 2 mmol/L were designed for the serum‐free starvation of cells for 6 h. Then, 10% complete medium was added to the blank group and 1 μg/mL LPS with 1% double antibody and 10% fetal bovine serum to the remaining concentrations of Ham's F 12K medium at 37°C and a 5% CO_2_ cell incubator for 48 h. Cells were collected for RNA extraction 48 h later.

### 2.4 Cell assay‐related experiments

#### 2.4.1 Determination of cell growth curves with the CCK‐8 method

The CCK‐8 method was employed to detect the viability of HFL1 cells. The mixture of cells transiently transfected in step 5 was added to a plate with 96 wells at 1 μL per well. Cells underwent 24‐, 48‐, and 72‐h transfection in the incubator. Each well was added with 10 μL of CCK‐8 reagent and experienced a further 2‐h culture in a 5% CO_2_ cell incubator at 37°C. The absorbance at the wavelength of 450 nm was gauged with an enzyme marker. The resulting data were recorded and saved. The viability (%) of cells was calculated as per the formula as follows: cell viability % = [A (spiked) – A (blank)]/[A (0 spiked) – A (blank)] × 100%.

#### 2.4.2 Fluorescent quantitative polymerase chain reaction (PCR)

The RT reaction solution was created using the TaKaRa TB Green® Premix Ex TaqTM II kit. PCR amplification was carried out using a steponeplus real‐time PCR apparatus. The 2‐△△Ct method was adopted to calculate the relative expression of the measured miRNA. The design and synthesis of all primers were completed by Sangong Bioengineering Technology Co., Ltd. (Shanghai, China), and primer sequences are displayed in Table 1.

**TABLE 1:** Primer sequences.

Primer name	Primer sequences (5′‐3′)
GAPDH‐F	GCCGCCCAGAACATCAT
GAPDH‐R	TGCCTGCTTCACCACCTT
TNFα‐F	AGCCCTGGTATGAGCCCATCTATC
TNFα‐R	TCCCAAAGTAGACCTGCCCAGAC
TGFβ1‐F	TACAGCAACAATTCCTGGCGATACC
TGFβ1‐R	CTCAACCACTGCCGCACAACTC
Smad7‐F	CTCGGAAGTCAAGAGGCTGTGTTG
Smad7‐R	TCTAGTTCGCAGAGTCGGCTAAGG
COL1A1‐F	TGATCGTGGTGAGACTGGTCCTG
COL1A1‐R	CTTTATGCCTCTGTCGCCCTGTTC

#### 2.4.3 Western blot

In this section, 30 mg of tumor tissue was weighed, and 300 μL of lysis solution (radio immunoprecipitation assay [RIPA]: phenylmethanesulfonyl fluoride (PMSF): protease inhibitor: phosphatase inhibitor = 100:1:1) was added. After lysis on ice, vortex and 15‐min centrifugation at 12 000 rpm at 4°C, the supernatant was taken, and the protein was quantified by the bicinchoninic acid (BCA) method. Next, 5× protein loading buffer was added to each sample, and equal mass and volume of each sample were made. The protein samples were boiled in boiling water at the temperature of 100°C for 10 min, spotted according to experimental requirements (internal reference sample volume: 30 ug, index sample volume: 30 ug) and subjected to sodium dodecyl sulphate‐polyacrylamide gel electrophoresis (SDS‐PAGE). Upon the completion of electrophoresis, the proteins on the protein gel were transferred to a polyvinylidene difluoride (PVDF) membrane and closed with 5% skimmed milk for 2 h. After closure, the samples were rinsed three times with TBS + Tween (TBST), followed by the dilution of corresponding antibodies according to the dilution ratio recommended in the antibody instruction. Corresponding antibodies were diluted. The primary antibody was incubated at 4°C overnight and rinsed three times with TBST. Next, the secondary antibody labeled with HRP underwent 1‐h incubation in a shaker at room temperature and three times washing with TBST. Electrochemiluminescence (ECL) chemiluminescence was developed. The gray value of target bands was calculated by Image Lab software, and the relative expression was determined by the following formula: relative amount of expression = target protein/internal reference protein.

### 2.5 Statistical analysis

Statistical analysis was performed using Statistical Package for the Social Sciences (SPSS) 22.0. Graphs were created using GraphPad Prism 9. Measures following normal distribution were indicated by mean ± standard deviation (x ± s). A comparison was made of multiple group means by analysis of variance (ANOVA) in a completely randomized design. A least significant difference (LSD)‐*t* test was utilized for two‐by‐two comparisons in the case of equal variances. A Dunnett's T3 test was conducted in the case of unequal variances. Statistically significant differences were found when *p* < 0.05. Flow cytometry experiments were analyzed with FlowJo 7.6.

## 3 RESULTS

### 3.1 Determination of PFD drug concentration

**FIGURE 1:**
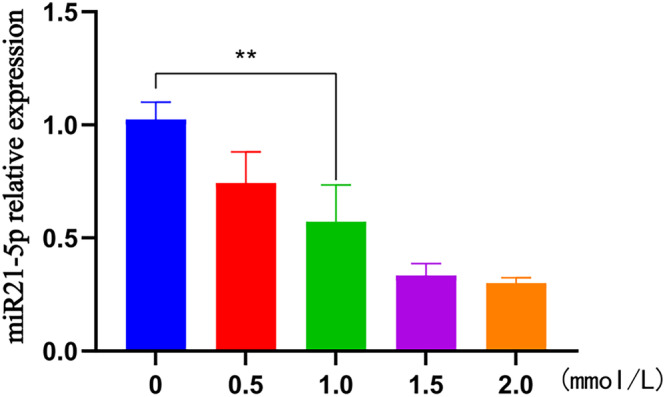
The h histogram of miR21‐5p relative expression in each group.

Fibroblasts were cultured for 48 h. The relative expressions of miR‐21‐5p in different concentration groups of 0, 0.5, 1, 1.5, and 2 mmol/L were 1.024 ± 0.076, 0.743 ± 0.137, 0.572 ± 0.162, 0.334 ± 0.053, and 0.301 ± 0.024, respectively. Compared with miR21‐5p in the concentration of 0 mmol/L, miR21‐5p in the other four concentration groups exhibited a down‐regulation in expression with statistically significant differences (*p* < 0.01). In addition, 1 mmol/L was selected as the intervention concentration of PFD in combination with cell growth status (Table 2, Figure 1).

**TABLE 2:** The miR21‐5p relative expression in each group (x̄ ± s).

Group	miR21‐5p
0 mmol/L	23.11 ± 0.11
0.5 mmol/L	23.58 ± 0.27^a^
1 mmol/L	24.83 ± 0.45^a^
1.5 mmol/L	25.09 ± 0.23^a^
2 mmol/L	24.365 ± 14.04^a^

^a^ All *p* are <0.01 compared with the 0 mmol/L control group.

### 3.2 Effect of PFD intervention with miR‐21‐5p on fibroblast proliferation

**FIGURE 2:**
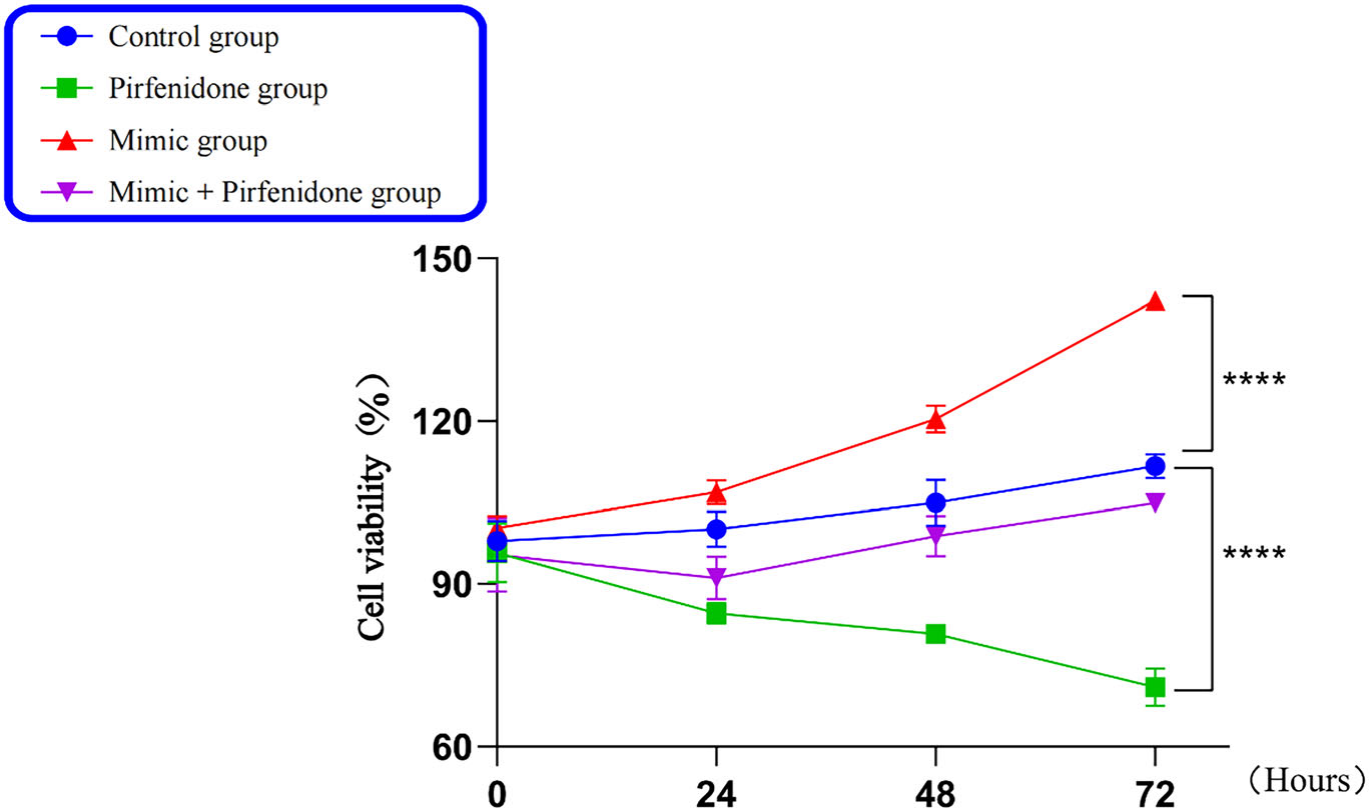
The growth curve of fibroblasts in each group.

The growth curves of fibroblasts at 0, 24, 48, and 72 h measured by CCK‐8 (Figure 2) showed that the mimic group exhibited the strongest cell viability of fibroblasts and the PFD group demonstrated the lowest cell viability of fibroblasts compared with the control one. The cell vitality of fibroblasts increased in the mimic + PFD group but decreased compared with that in the mimic one. This indicates that PFD reverses the proliferative effect of miR‐21‐5p on fibroblast promotion.

### 3.3 Relationships between PFD, miR‐21‐5p, and inflammation and fibrosis‐related factors

**FIGURE 3:**
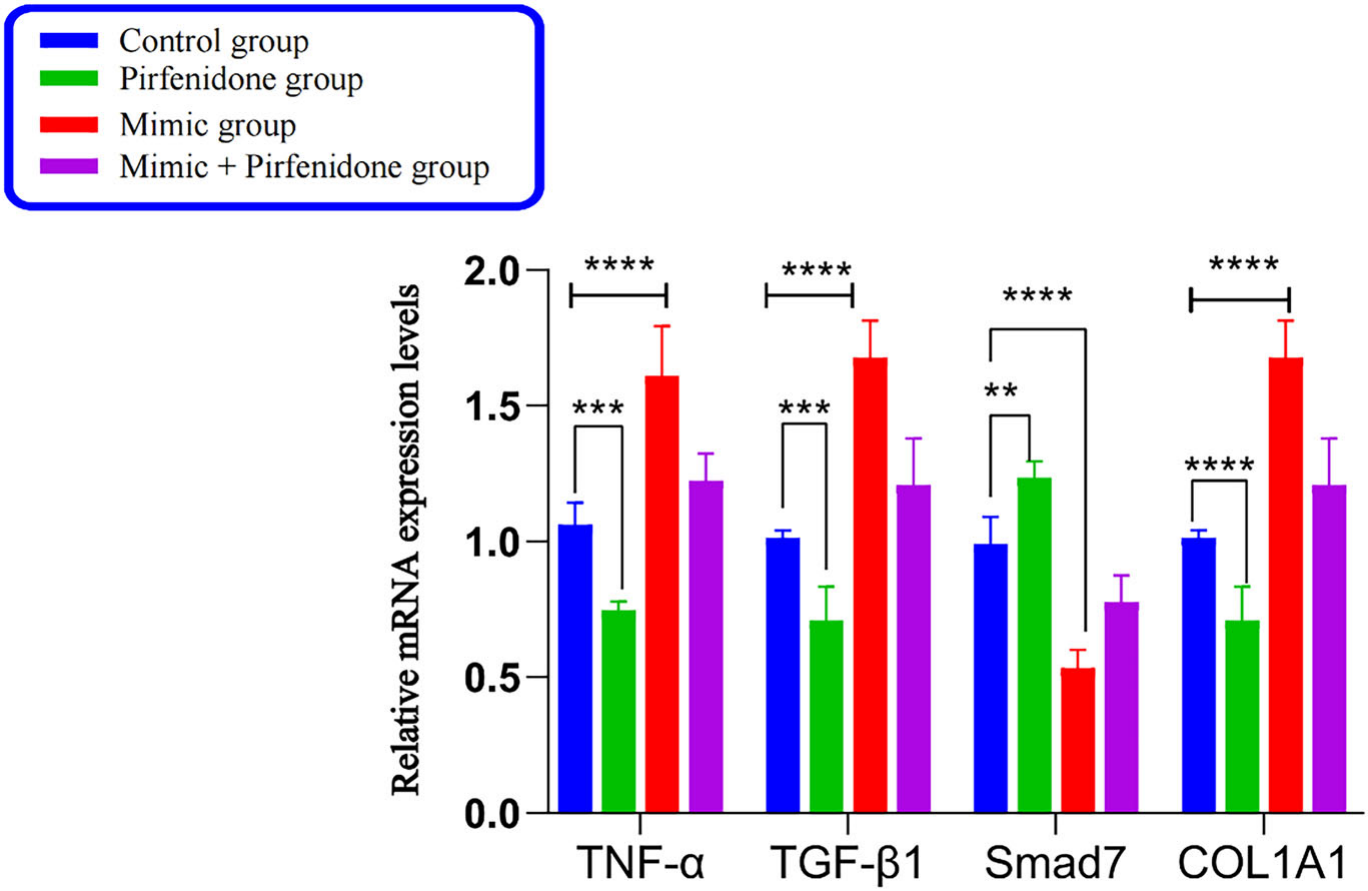
The histogram of relative mRNA expression in each group. *Note*: TNF‐α, TGF‐β1, and COL1A1mRNA were up‐regulated, and Smad7mRNA was down‐regulated in the mimic group. The expression of mRNA of the above correlated factors is opposite in the pirfenidone group.

**FIGURE 4:**
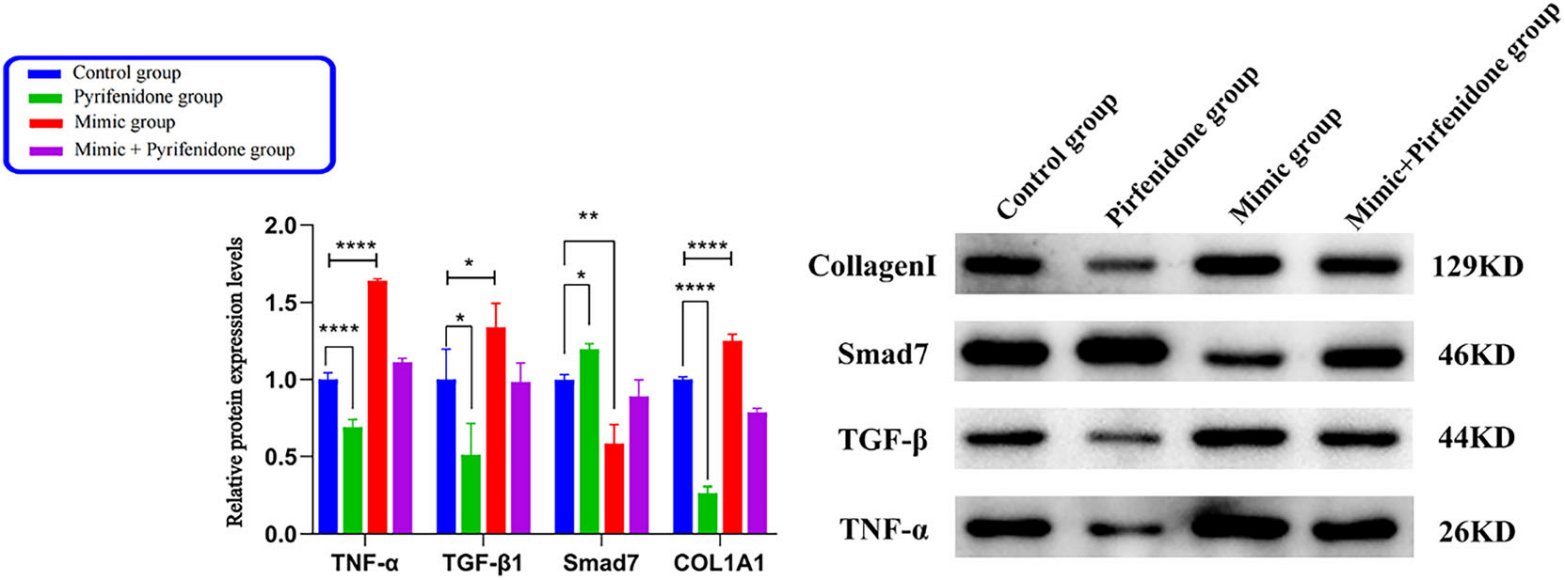
The histogram of relative protein expression in each group. *Note*: TNF‐α, TGF‐β1, and COL1A1 protein were up‐regulated, and Smad 7 protein was downregulated in the mimic group; the expression of the above related factor proteins in the pirfenidone group was reversed.

1. The following real‐time quantitative polymerase chain reaction (RT‐qPCR) results (Table 3, Figure 3) showed: the mimic group exhibited an up‐regulation in the relative expressions of tumor necrosis factor‐α (TNF‐α), transforming growth factor‐β1 (TGF‐β1), and collagen type I alpha 1 messenger RNA (COL1A1 mRNA) and a down‐regulation in the relative expression of drosophila mothers against decapentaplegic protein 7 (Smad7) mRNA compared with the control one. The results of the factors mentioned above were reversed in the PFD group. Despite the up‐regulation in the relative expressions of TNF‐α, TGF‐β1, and COL1A1 mRNA and the down‐regulation of Smad7 mRNA in the mimic + PFD group, the degree of up‐regulation or down‐regulation was smaller than that of the mimic group. This implies that PFD inhibits the pro‐inflammatory and ‐fibrotic actions of miR‐21‐5p.
2. The following WB results (Table 4, Figure 4) showed: the mimic group exhibited a significant up‐regulation in the relative expressions of TNF‐α, TGF‐β1, and COL1A1 proteins and the down‐regulation of Smad7 protein compared with the control one. The results of the factors mentioned above were reversed in the PFD group. Despite the up‐regulation in the relative expressions of TNF‐α, TGF‐β1, and COL1A1 and the down‐regulation of Smad7 in the mimic + PFD group, the degree of up‐regulation or down‐regulation was smaller than that of the mimic group. This suggests that PFD inhibits the pro‐inflammatory and ‐fibrotic actions of miR‐21‐5p.

**TABLE 3:** The relative expression of mRNA in each group (x̄ ± s).

Group	Control group	Pyrifenidone group	Mimic group	Mimic + Pyrifenidone group
TNF‐α	1.062 ± 0.083	0.747 ± 0.033^a^	1.612 ± 0.182^a^	1.226 ± 0.101
TGF‐β1	1.016 ± 0.026	0.708 ± 0.126^a^	1.677 ± 0.138^a^	1.209 ± 0.170
Smad7	0.991 ± 0.100	1.236 ± 0.060^a^	0.535 ± 0.067^a^	0.778 ± 0.098
COL1A1	1.016 ± 0.027	0.708 ± 0.126^a^	1.677 ± 0.138^a^	1.209 ± 0.170

^a^ *p* < 0.01 as compared with the control group.

**TABLE 4:** The relative protein expression in each group (x̄ ± s).

Group	Control group	Pyrifenidone group	Mimic group	Mimic + Pyrifenidone group
TNF‐α	1.000 ± 0.044	0.691 ± 0.050^a^	1.640 ± 0.011^a^	1.112 ± 0.026
TGF‐β1	1.000 ± 0.197	0.511 ± 0.207^a^	1.340 ± 0.155^a^	0.984 ± 0.124
Smad7	0.999 ± 0.034	1.197 ± 0.035^a^	0.584 ± 0.122^a^	0.891 ± 0.105
COL1A1	1.000 ± 0.018	0.267 ± 0.040^a^	1.253 ± 0.041^a^	0.786 ± 0.027

^a^ *p* < 0.01 as compared with the control group.

## 4 DISCUSSION

With the rapid development of critical care medicine, tracheal intubation or tracheotomy has been widely used in clinical practice. It may also result in ATS despite saving the lives of numerous severely ill patients. That is the main factor causing complex and difficult airway stenosis. A retrospective observational study of 357 patients receiving prolonged mechanical ventilation showed that 272 patients (76%) underwent percutaneous tracheostomies, and 114 (32%) presented mild‐to‐moderate tracheal stenosis after weaning completion. A majority of stenoses (88%) were situated in the upper trachea and most commonly attributed to the formation of localized granulation tissues at the internal stoma site (96%).^15^ The management of ATS is limited in therapeutic options. Endoscopy, a minimally invasive approach, is very likely to cause the recurrence of stenosis. Some topical agents including steroid injections, topical heparin, topical mitomycin C (MMC), and 5‐fluorouracil^16^ have been utilized as adjuvants in endoscopic treatment. However, uniform conclusions have not been drawn yet, and these topical agents have certain toxic side effects. In 2020, a systematic review was made of experimental or observational research that has used topical MMC to treat laryngotracheal stenosis. Evidence suggests that MMC is a safe and effective option in the endoscopic therapy of laryngotracheal stenosis.^17^ In 2023, however, Felice et al.^18^ reviewed and synthesized the existing literature on MMC as an adjunctive therapy for laryngotracheal stenosis. Finally, they found that the role of MMC as an adjunctive therapy in LTS is not certain. Notwithstanding its safe application, MMC still triggers controversies over its efficacy in reducing stenosis recurrence. It is necessary to propose future recommendations by conducting large prospective studies. Therefore, how to inhibit or reverse the formation of tracheal scars is an important topic of medical research today.

The pathogenesis of tracheal stenosis has revealed that the injured tracheas are pathologically repaired during the healing process. This involves numerous complex pathophysiological changes. These changes are inflammatory responses, cell differentiation and proliferation, matrix deposition, primarily fibroblast‐based cell overproliferation, the massive accumulation of type I and type III collagen‐based ECMs, the proliferation of tracheal scar tissues, and so on. Eventually, tracheal stenosis is caused. PFD has been studied for usage in harmful tracheal scar stenosis^19^ owing to its broad anti‐fibrotic, ‐inflammatory, and ‐oxidant actions with moderate success. For instance, Türkmen and Pata^20^ performed tracheotomies on 14 rats and secured the tracheal cannula there using a stoma suture. The experimental group received PFD 15 mg/kg/day intraperitoneally for 10 days, whereas the control one was given a 1‐mL intraperitoneal injection of saline. Tracheal intubation was removed after 10 days, and the rats were executed on Day 30. The trachea was excised between the first and seventh rings. Histopathology was used to identify tracheal mucosal damage, inflammation, and scar fiber formation. In comparison with the control group, this group exhibited an improvement in the extent of scar stenosis following pneumonectomy treated with PFD. PFD might inhibit the expressions of TGF‐α, TNF‐β1, and interleukin‐1β (IL‐1β); diminish inflammatory responses and fibrotic effects; and alleviate scar stenosis. The study by Shi et al.^21^ on human epidural scar fibroblasts demonstrated that PFD inhibited the phosphorylation of Smad2, Smad3, protein kinase B (Akt), and p38 induced by TGF‐β1, reduced α‐smooth muscle actin (α‐SMA) production and collagen synthesis, and curbed the proliferation of human epidural scar fibroblasts. Thus, applying PFD topically could reduce epidural scar fibrosis formation, scar adhesions, and other complications after laminectomy. Moreover, Li et al.^22^ were the first to use PFD for treating secondary scarring tracheobronchial stenosis in two cases. It was concluded that the combination of PFD and endoscopic intervention is likely to effectively relieve tracheobronchial stenosis induced by trauma.

In this section, the fibroblast generation curves at 0, 24, 48, and 72 h were observed by CCK‐8, respectively, in the experiment of LPS‐induced fibroblast proliferation by PFD intervention in the transfection of miR‐21‐5p. This indicates that the fibroblast growth curve in the PFD group showed a continuous decrease, while the fibroblasts in the miR‐21‐5p mimic group demonstrated a continuous upward trend of growth. At 72 h, PFD decreased fibroblast viability from 91% to 82% compared with that in the control group, which showed that PFD inhibited the viability of fibroblasts and affected their proliferation. However, miR‐21‐5p increased fibroblast viability from 91% to 120%, which suggested that miR‐21‐5p promoted fibroblast proliferation. The miR‐21‐5p + PFD group showed a fibroblast viability of 85%, which demonstrated that PFD inhibited the effect of miR‐21‐5p on the promotion of fibroblast proliferation. RT‐qPCR and WB were used to detect the expressions of TNF‐α, TGF‐β1, COL1A1, and Smad7 mRNA and proteins in each group. The PFD group showed a decrease in the expressions of TNF‐α, TGF‐β1, and COL1A1 mRNA and proteins but an increase in the expression of Smad7 mRNA and protein compared with the control one. This indicates that PFD had anti‐inflammatory and ‐fibrotic effects. The miR‐21‐5p group exhibited an up‐regulation in the expressions of TNF‐α, TGF‐β1, and COL1A1 mRNA and proteins but a down‐regulation in the expression of Smad7 mRNA and protein. This is consistent with the previous study, which showed that miR‐21‐5p could promote inflammatory and fibrotic responses. The miR‐21‐5p + PFD group demonstrated an increase in the expressions of TNF‐α, TGF‐β1, and COL1A1 mRNA and proteins and a down‐regulation in the expression of Smad7 mRNA and protein but a lesser extent of down‐regulation than the miR‐21‐5p mimic one. This suggests that PFD can attenuate the pro‐inflammatory and ‐fibrotic responses of miR‐21‐5p. Then, what is the underlying molecular mechanism? MiR‐21‐5p has been demonstrated to directly bind to Smad7, affect its post‐transcriptional expression, and improve the transduction of the TGF‐β/Samds signaling pathway. Meanwhile, it can cause high levels of the TGF‐β1‐mediated associated fibrotic factor COL1A1 and the inflammatory factor TNF‐α, intensify inflammatory and fibrotic responses, and thereby promote fibroblast proliferation. According to this section of the experimental findings, PFD can suppress miR‐21‐5p, which may promote fibroblast proliferation and have pro‐inflammatory and ‐fibrotic effects. Thus, it was hypothesized that PFD may inhibit TGF‐β1‐mediated fibrosis and inflammation by down‐regulating miR‐21‐5p and up‐regulating Smad7 expression and finally restrain fibroblast proliferation and fibrosis progression. In agreement with similar findings, Escutia‐Gutiérrez et al.^23^ discovered the significant expression of miR21a‐5p in C57BL/6J mice with metabolic‐associated fatty liver disease, along with the up‐regulation of inflammatory factors IL‐1, IL‐6, IL‐8, and TNF‐α; fibrogenic factors TGF‐β1 and α‐SMA; collagen genes; and protein levels. In mouse blood after PFD treatment, a significant drop occurred in miR21a‐5p expression and associated inflammatory and fibrogenic components. Collagen and α‐SMA levels in liver tissue sections declined significantly as well, and the activity of stellate cells was inhibited. The results suggest that PFD stops the progression of fatty liver disease to fibrosis by down‐regulating miR21a‐5p and its mediated expression of inflammatory and fibrotic factors, which thereby inhibits inflammatory responses and ameliorates fibrotic effects. Bi^24^ discovered that the renal tissues of miR‐21, TGF‐β1, Smad3, collagen III and E‐calmodulin, α‐SMA genes, and protein levels were up‐regulated in the Sprague Dawley rat model of renal tubular interstitial fibrosis, whereas Smad7 was down‐regulated. In vitro cellular experiments demonstrated that the over‐expression of miR‐21 and the down‐regulation of Smad7 boosted the buildup of epithelial‐mesenchymal transition (EMT) and ECMs. Following that, in vitro and in vivo dual model experiments with PFD intervention revealed that PFD alleviated tubulointerstitial fibrosis in unilateral ureteral obstruction (UUO) rats by down‐regulating miR‐21 expression and up‐regulating Smad7 expression, which ultimately inhibited the activation of the TGF‐β1/Smad3 signaling pathway.

## 5 CONCLUSION

The experimental findings of this study suggest that PFD may have a preventive and curative effect on harmful tracheal stenosis by inhibiting fibroblast proliferation and the fibrotic process and possibly through down‐regulating miR‐21‐5p and up‐regulating Smad7 and its mediated fibrotic and inflammatory responses. However, some limitations still exist in this experimental study: first, this study is an in vitro cell experiment and lacks in vivo animal experimental research and the exploration of whether the changes of relevant indicators are consistent with in vivo experiments. Second, the mechanism of interactions between PFD, miR‐21‐5p, and Smad7 is unclear and whether miR‐21‐5p inhibits fibroblast proliferation by targeting Smad7 lacks a dual luciferase reporter system. These two parts will be further elaborated and demonstrated in subsequent experiments, to provide a strong experimental basis for treating ATS.

## AUTHOR CONTRIBUTIONS

Analyzed data and wrote the paper: Wentao Li; Pingping Huang. Designed the study: Jinmei Wei. Collected and analyzed data: Sen Tan, Qiu Yang. Designed the study and reviewed the manuscript: Guangnan Liu, Guangfa Wang. All authors have read and approved the final manuscript.

## CONFLICT OF INTEREST STATEMENT

No potential conflict of interest was reported by the authors.

## ETHICS STATEMENT

The Ethics Committee of the Second Affiliated Hospital of Guangxi Medical University, in Nanning, China, gave its approval for this study's conduct, which was carried out in accordance with the guidelines specified in the Declaration of Helsinki.

## Data Availability

The data used for supporting the findings of this study are available from the corresponding authors upon request.
